# The Role of Deleterious Substitutions in Crop Genomes

**DOI:** 10.1093/molbev/msw102

**Published:** 2016-06-14

**Authors:** Thomas J. Y. Kono, Fengli Fu, Mohsen Mohammadi, Paul J. Hoffman, Chaochih Liu, Robert M. Stupar, Kevin P. Smith, Peter Tiffin, Justin C. Fay, Peter L. Morrell

**Affiliations:** ^1^Department of Agronomy and Plant Genetics, University of Minnesota; ^2^Department of Agronomy, Purdue University; ^3^Department of Plant Biology, University of Minnesota; ^4^Department of Genetics, Washington University

**Keywords:** crops, deleterious mutations, resequencing, bioinformatics.

## Abstract

Populations continually incur new mutations with fitness effects ranging from lethal to adaptive. While the distribution of fitness effects of new mutations is not directly observable, many mutations likely either have no effect on organismal fitness or are deleterious. Historically, it has been hypothesized that a population may carry many mildly deleterious variants as segregating variation, which reduces the mean absolute fitness of the population. Recent advances in sequencing technology and sequence conservation-based metrics for inferring the functional effect of a variant permit examination of the persistence of deleterious variants in populations. The issue of segregating deleterious variation is particularly important for crop improvement, because the demographic history of domestication and breeding allows deleterious variants to persist and reach moderate frequency, potentially reducing crop productivity. In this study, we use exome resequencing of 15 barley accessions and genome resequencing of 8 soybean accessions to investigate the prevalence of deleterious single nucleotide polymorphisms (SNPs) in the protein-coding regions of the genomes of two crops. We conclude that individual cultivars carry hundreds of deleterious SNPs on average, and that nonsense variants make up a minority of deleterious SNPs. Our approach identifies known phenotype-altering variants as deleterious more frequently than the genome-wide average, suggesting that putatively deleterious variants are likely to affect phenotypic variation. We also report the implementation of a SNP annotation tool BAD_Mutations that makes use of a likelihood ratio test based on alignment of all currently publicly available Angiosperm genomes.

## Introduction

Mutation produces a constant influx of genetic variants into populations. Each mutation has a fitness effect that varies from lethal to neutral to advantageous. While the distribution of fitness effects of new mutations is not directly observable (Eyre-Walker and Keightley 2007), most mutations with fitness impacts are deleterious (Keightley and Lynch 2003). It is generally assumed that deleterious mutations alter phylogenetically conserved sites (Doniger et al. 2008), or cause loss of protein function (Yampolsky et al. 2005). Strongly deleterious mutations (particularly those with dominant effects) are quickly purged from populations by purifying selection. Similarly, strongly advantageous mutations increase in frequency, and ultimately fix due to positive selection (Robertson 1960; Smith and Haigh 1974). Weakly deleterious mutations have the potential to persist in populations and cumulatively contribute significantly to reductions in fitness as segregating deleterious variants (Fay et al. 2001; Eyre-Walker et al. 2006; Doniger et al. 2008).

Considering a single variant in a population, three parameters affect its segregation: the effective population size (*N*_e_), the selective coefficient against homozygous individuals (*s*), and the dominance coefficient (*h*). The effects of *N*_e_ and *s* are relatively simple: variants are primarily subject to genetic drift rather than selection if the product of negative selective coefficients and *N*_e_ is less than 1, that is, (*N*_e_*s*) < 1 (Kimura et al. 1963). The effect of *h* is not as straightforward, as it depends on the genotypic frequencies and the degree of outcrossing in the population. In populations with a high degree of self-fertilization or sibling mating, many individuals will be homozygous, which reduces the importance of *h* in determining the efficacy of selection against the variant (Glémin 2003). In populations that are closer to panmixia, an individual deleterious variant will occur primarily in the heterozygous state, and *h* will determine how “visible” the variant is to selection, with higher values of *h* increasing the efficacy of selection (Charlesworth and Charlesworth 1999). A completely recessive deleterious variant may remain effectively neutral as long as the frequency of the variant is low enough such that there are not a substantial number of homozygous carriers. Conversely, a completely dominant deleterious variant is expected to be quickly purged from the population (Lande and Schemske 1985). On average, deleterious variants segregating in a population are predicted to be partially recessive (Simmons and Crow 1977), allowing them to remain “hidden” from the action of purifying selection, and reach moderate frequencies. This may be expected, for example, based on data from a gene knockout library in yeast (Shoemaker et al. 1996), which indicate that protein loss-of-function variants have an average dominance coefficient of 0.2 (Agrawal and Whitlock 2012).

Effective recombination rate also has important impacts on the number and distribution of deleterious mutations in the genome. Regions with low effective recombination are prone to the irreversible accumulation of deleterious variants. This phenomenon is known as the “ratchet effect” (Muller 1964). In finite populations with low recombination, the continual input of deleterious mutations and stochastic variation in reproduction causes the loss of individuals with the fewest deleterious variants. Lack of recombination precludes the selective elimination of chromosomal segments carrying deleterious variants, and thus they can irreversibly increase, similar to how a ratchet turns in only one direction (Muller 1964). Nordborg (2000) demonstrates that under high levels of inbreeding, effective recombination rate can be decreased by almost 20-fold relative to an outbreeding population, showing that mating system can be a major determinant in the segregation of deleterious variation. While inbreeding populations are especially susceptible to ratchet effects on a genome-wide scale, even outbreeding species have genomic regions with limited effective recombination (Arnheim et al. 2003; McMullen et al. 2009). In maize, these low recombination regions are observed to harbor excess heterozygosity in inbred lines, suggesting that they maintain deleterious variants that cannot be made homozygous (Rodgers-Melnick et al. 2015). Both simulation studies (Felsenstein 1974) and empirical investigations in *Drosophila melanogaster* (Campos et al. 2012, 2014) indicate that deleterious variants accumulate in regions of limited recombination.

Efforts to identify deleterious variants and quantify them in individuals have led to a new branch of genomics research. In humans, examination of the contribution of rare deleterious variants to heritable disease has contributed to the emergence of personalized genomics as a field of study (reviewed in Abecasis et al. 2010; Cooper et al. 2010; Marth et al. 2011). Current estimates suggest that an average human may carry ∼300 loss-of-function variants (Abecasis et al. 2010; Agrawal and Whitlock 2012) and up to tens of thousands of weakly deleterious variants in coding and functional noncoding regions of the genome (Arbiza et al. 2013). In terms of effects on organismal fitness, the average human carries three lethal equivalents (Gao et al. 2015; Henn et al. 2015). These variants are enriched for mutations that are causative for diseases (Kryukov et al. 2007; Marth et al. 2011). As such they are expected to have appreciable *N*_e_*s* and be kept at low frequencies due to the action of purifying selection.

Humans are not unique in harboring substantial numbers of deleterious variants. It is estimated that almost 40% of nonsynonymous variants in *Saccahromyces cerevisiae* have deleterious effects (Doniger et al. 2008) and 20% of nonsynonymous variants in rice (Lu et al. 2006), *Arabidopsis thaliana* (Günther and Schmid 2010), and maize (Mezmouk and Ross-Ibarra 2014) are deleterious. In dogs, Cruz et al. (2008) identified an excess of nonsynonymous single nucleotide polymorphisms (SNPs) segregating in domesticated dogs relative to grey wolves. A similar pattern has been found in horses (Schubert et al. 2014) and sunflowers (Renaut and Rieseberg 2015), suggesting that an increased prevalence of deleterious variants may be a “cost of domestication.”

Approaches to identify deleterious mutations take one of two forms. Quantitative genetic approaches have been employed that investigate the aggregate impact of potentially deleterious alleles on fitness. Mutation accumulation studies (e.g., Mukai 1964; Schultz et al. 1999; Shaw et al. 2002; Charlesworth et al. 2004) use change in fitness over generations within lineages to estimate mutational effects on fitness. Coupled with DNA sequencing technologies, these studies may shed light on how many DNA sequence changes are potentially deleterious (e.g., Ossowski et al. 2010). On the other hand, purely bioinformatic approaches make use of measures of sequence conservation to identify variants with a significant probability of being deleterious. When combined with genome-scale resequencing, they permit the identification of large numbers of putatively deleterious variants. Commonly applied approaches include sorting intolerant from tolerated (SIFT) (Ng 2003), PolyPhen2 (Polymorphism Phenotyping) (Adzhubei et al. 2010), and a likelihood ratio test (LRT) (Chun and Fay 2009). These sequence conservation approaches operate in the absence of phenotypic data, but allow assessment of individual sequence variants. As such, some variants identified bioinformatically may be locally adaptive, or conditionally neutral. However, given the observation that deleterious mutations constantly arise and continue to segregate in populations, their targeted identification and elimination from breeding populations presents a novel path for crop improvement (Morrell et al. 2011).

In this study, we investigate the distribution of deleterious variants in 13 barley (*Hordeum vulgare* ssp. *vulgare*) cultivars, two wild barley (*H. vulgare* ssp. *spontaneum*) accessions, seven soybean (*Glycine max*) cultivars, and one wild soybean (*Glycine soja*) accession using exome and whole genome resequencing, respectively. We seek to answer four questions about the presence of deleterious variants: (1) How many deleterious variants do individual cultivars harbor, and what proportion of these are nonsense (early stop codons) versus nonsynonymous (missense) variants? (2) What proportion of nonsynonymous variation is inferred to be deleterious? (3) How many known phenotype-altering SNPs are inferred to be deleterious? (4) How does the relative frequency of deleterious variants vary with recombination rate? We identify an average of ∼1,000 deleterious variants per accession in our barley sample and ∼700 deleterious variants per accession in our soybean sample. Approximately 40% of the deleterious variants are private to one individual in both species, suggesting the potential for selection for individuals with a reduced number of deleterious variants. Approximately 3–6% of nonsynonymous variants are inferred to be deleterious by all three annotation approaches used in our study, and known causative SNPs annotate as deleterious at a much higher proportion than the genomic average. In soybean, where genome-wide recombination rate estimates are available, the proportion of deleterious variants is negatively correlated with recombination rate.

## Results

### Variant Calling and Identification of Deleterious SNPs

Resequencing and read mapping followed by read de-duplication resulted in an average coverage of ∼39X exome coverage for our barley samples and ∼38X genome coverage in soybean. A summary of our resequencing data and read mapping statistics is shown in supplementary table S1, Supplementary Material online. Average heterozygosity was 2.5% in our barley sample, and 0% in our soybean sample, reflecting the inbreeding of the accessions. The observed heterozygosity in our barley sample is mostly due to the inclusion of wild material, which is less inbred than the cultivars. Heterozygous variant calls in soybean were all in reads with low mapping score, possibly due to the highly duplicated nature of the soybean genome (Schmutz et al. 2010). A table of the barley accessions used in this study is shown in supplementary table S2, Supplementary Material online, and the soybean accessions are shown in supplementary table S3, Supplementary Material online. All analyses reported here are performed on SNPs.

**Fig. 1.**
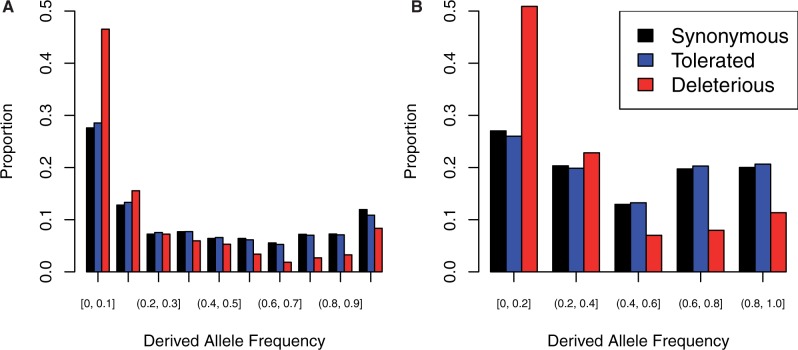
Derived allele (unfolded) frequency spectra for coding regions showing deleterious, tolerated, and synonymous SNPs for barley and soybean. Ancestral state was inferred as described in the Methods. A variant was called “Deleterious” if it was nonsynonymous and predicted to be deleterious by SIFT, PolyPhen2, and the LRT. (*A*) is based on 13 domesticated barley accessions and 2 wild accessions while (*B*) is based on seven cultivated soybean accessions and one wild accession.

After realignment and variant recalibration, we identified 652,797 SNPs in 13 cultivated and 2 wild barley lines. The majority of these SNPs were noncoding, with 522,863 occurring outside of coding sequence (CDS) annotations. Of the coding SNPs, 70,069 were synonymous, and 59,865 were nonsynonymous. A summary of the variants in various functional classes is shown in table 1, and a per-sample summary is shown in supplementary table S4, Supplementary Material online. SIFT identified 13,626 SNPs as deleterious, PolyPhen2 identified 13,534 SNPs to be deleterious, and the LRT called 17,865 deleterious. The intersection of all three approaches identifies a much smaller set of deleterious variants, with a total of 4,872 nonsynonymous SNPs identified as deleterious. While individual methods identified ∼18% of nonsynonymous variants as deleterious, the intersect of approaches identifies 5.7%. A derived allele frequency spectrum of synonymous, nonsynonymous, and putatively deleterious SNPs in our barley sample is shown in fig. 1*A*.

**Table 1:** Mean Numbers of SNPs in Various Classes.

Species	Diff. from Ref.	Noncoding	Syn.	Nonsyn.	Nonsense
Barley	162,954 (51,231.34)	115,456 (41,065.22)	15,591 (5,691.81)	12,351 (4,492.53)	77 (33.13)
Soybean	82,840 (56,780.03)	44,704 (29,477.65)	14,167 (8,161.21)	18,695 (11,289.72)	540 (345.05)

Syn., Synonymous; Nonsyn., Nonsynonymous. Numbers are mean (SD)

**Fig. 2.**
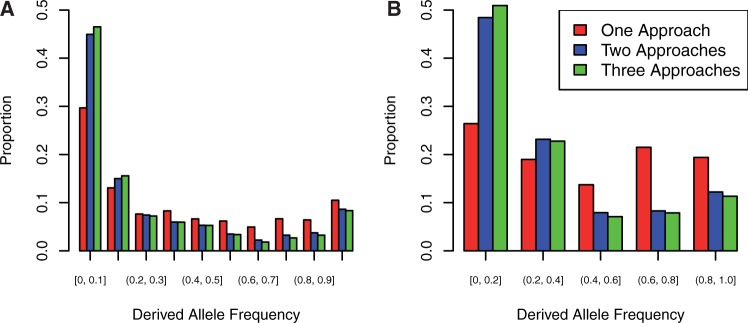
Derived allele (unfolded) frequency spectra for SNPs in (*A*) barley and (*B*) soybean predicted to be deleterious by one, two, or three prediction approaches. SNPs predicted by only one approach are not as strongly skewed toward rare variants, suggesting that the intersection of multiple prediction approaches gives the most reliable prediction of deleterious variants.

In soybean, we called 586,102 SNPs in gene regions. Of these, 542,558 occurred in the flanking regions of a gene model. We identified 73,577 synonymous SNPs, and 99,685 nonsynonymous SNPs (supplementary table S5, Supplementary Material online). SNPs in the various classes sum to greater than the total number of SNPs as a single SNP in multiple transcripts can have multiple functional annotations. For instance, a SNP may be intronic in one transcript, but exonic in an alternative transcript. SIFT identified 7,694 of the nonsynonymous SNPs as deleterious, PolyPhen2 identified 14,933 as deleterious, and the LRT identified 11,223 as deleterious. Per-sample counts of putatively deleterious variants in barley are shown in supplementary table S6, Supplementary Material online, and per-sample counts for soybean are shown in supplementary table S7, Supplementary Material online. Similar to the barley sample, the proportion of putatively deleterious SNPs was similar across prediction approaches, with the exception of SIFT, which failed to find alignments for many genes. The overlap of prediction approaches identified 3,041 (2.6%) of nonsynonymous SNPs to be deleterious (table 2). Derived allele frequency distributions are shown in fig. 1*B*. SNPs inferred to be deleterious are generally at lower derived allele frequency than other classes of variation, implying that these SNPs are truly deleterious. For both species, the intersection of approaches appeared to give the most accurate prediction of whether or not a SNP is deleterious, as evidenced by enrichment for rare alleles (fig. 2).

**Table 2:** Mean Counts of Nonsynonymous Variants That Are Predicted to Be Deleterious by Three Prediction Methods.

Species	SIFT	PPH	LRT	Intersect
Barley	3,400 (0.192)	3,295 (0.186)	3,221 (0.183)	1,006 (0.057)
Soybean	1,972 (0.064)	3,881 (0.126)	3,135 (0.101)	784 (0.025)

Numbers in parentheses are proportions of all nonsynonymous variants in each sample that are predicted to be deleterious.

Nonsense SNPs made up a relatively small proportion of putatively deleterious SNPs. In our barley sample, we identified a total of 711 nonsense SNPs, 14.5% of our putatively deleterious SNPs. In soybean, we identified 1,081 nonsense SNPs, which make up 15.7% of putatively deleterious SNPs. Nonsense SNPs have a higher heterozygosity than tolerated, silent, or deleterious missense SNPs (supplementary fig. S1, Supplementary Material online). While the absolute differences in heterozygosity were small due to the inbred nature of our samples, the pattern suggests that nonsense SNPs are more strongly deleterious than missense SNPs.

The transition to transversion ratio in our barley samples was 1.7:1 (supplementary fig. S2*B*, Supplementary Material online), very close to estimates obtained from previous Sanger resequencing in barley genes (Morrell et al. 2006). In soybean, the transition to transversion ratio in our SNPs was 1.4:1, while the estimate from a Sanger resequencing dataset was ∼1.2:1 (Hyten et al. 2006). The differences we observe between results from Sanger and Illumina resequencing could be due to the duplicated nature of the soybean genome (Schmutz et al. 2010), leading to paralogous alignment.

### Deleterious Mutations and Causative Variants

Bioinformatic approaches for identifying deleterious SNPs rely on sequence constraint to estimate protein functional impact. An example of a deleterious SNP showing a derived base substitution that alters a phylogenetically conserved codon is shown in supplementary fig. S2, Supplementary Material online. The SNPs identified in these approaches should be enriched for SNPs that cause large phenotypic changes. To explore how frequently known causative SNPs annotate as deleterious, we compiled a list of 23 nonsynonymous SNPs inferred to contribute to known phenotypic variation in barley and 11 in soybean, and tested the effect of these SNPs in our prediction pipeline. Of 23 SNPs that are purported to be causative for large phenotypic changes, 6 (25%) were inferred to be deleterious (supplementary table S8, Supplementary Material online). Of the 11 soybean putatively causative SNPs, 5 (45%) were inferred to be deleterious. This contrasts with the genome-wide average of ∼3–6%, showing that SNPs our pipeline identifies as deleterious are more likely to impact phenotypes.

### Deleterious Mutations and Genetic Map Distance

**Fig. 3.**
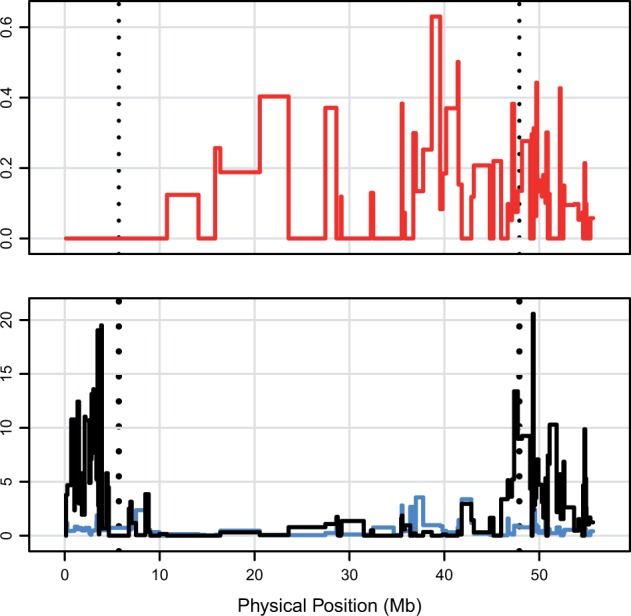
Comparison between recombination rate, CDS diversity, and proportion of nonsynonymous SNPs inferred to be deleterious in soybean on chromosome 1. The top panel shows the proportion of nonsynonymous SNPs that were inferred to be deleterious, in windows defined by genetic map distance (Lee et al. 2015). The bottom panel shows recombination rate in cM/Mb (black line) and average pairwise nucleotide sequence diversity per kilobase in CDS (blue line). Dashed vertical lines represent the boundaries of the annotated pericentromeric region, which has much lower recombination rates than the euochromatic regions.

The effective recombination rate strongly influences the purging of deleterious variants from populations. To examine the relationship between the number of deleterious SNPs and recombination rate, we used a high-density genetic map from a soybean recombinant inbred line family (Lee et al. 2015). The soybean map was based on a subset of the SoySNP50K genotyping platform (Song et al. 2013). There was a weak yet significant correlation between recombination rate and the proportion of nonsynonymous SNPs inferred to be deleterious (*r*^2 ^=^ ^0.007, *P* < 0.001, fig. 3 and supplementary S3, Supplementary Material online). We did not examine this relationship in barley because the barley reference genome assembly (Mayer et al. 2012) contains limited physical distance information.

## Discussion

Questions regarding the prevalence of deleterious variants date back over half a century (Fisher 1930; Muller 1950). In finite populations, the segregation of deleterious variants can have a substantial impact on population mean fitness (Kimura et al. 1963). While it has been argued that the concept of a reduction of fitness relative to a hypothetical optimal genotype is irrelevant (Wallace 1970), mutation accumulation studies have shown that new mutations have a substantial effect on absolute fitness (Schultz et al. 1999; Shaw et al. 2002).

Our results demonstrate that a large number of putatively deleterious variants persist in individual cultivars in both barley and soybean. The approaches used in this study predict the probability that a given amino acid or nucleotide substitution disrupts protein function. Mutations that alter phenotypes may be especially likely to annotate as deleterious, and we show that a high proportion of inferred causative mutations annotate as deleterious. It should be noted that variants identified as deleterious may affect a phenotype that is adaptive in only part of the species range or has a transient selective advantage—that is, locally or temporally adaptive phenotypes. Our panel of causative variants consists primarily of SNPs that confer an agronomically important phenotype (supplementary table S5, Supplementary Material online). Agronomic phenotypes may be beneficial in wild populations, particularly biotic and abiotic stress tolerance or reproductive traits (Mercer et al. 2007), but are not expected to be either globally deleterious or globally beneficial. If the portion of the range in which the phenotype is adaptive is small or the selective advantage is transient, such variants will be kept at low frequencies and also be identified as deleterious. Just as few variants are expected to be globally advantageous, a portion of deleterious variation is likely to not be globally deleterious. Such variants could be either locally or temporally advantageous, with a fitness advantage under some circumstances contributing to their maintenance in populations (Tiffin and Ross-Ibarra 2014).

At the molecular level, variants occurring in minor transcripts of genes may exhibit conditional neutrality (Tiffin and Ross-Ibarra 2014), and *N*_e_*s* will be too low for purifying selection to act. Gan et al. (2011) identified many isoforms of genes among a diverse panel of *A.* *thaliana* accessions, as well as compensatory mutations for a majority of frameshift mutations. Genetic variants that annotated as nonsynonymous or nonsense using the *A. thaliana* reference are frequently spliced out of the transcript such that the gene still produces a full-length and functional product. In a similar vein, deleterious variants may have their fitness impacts offset by compensatory mutations. In a study of bacteriophage, ∼70% of deleterious mutations were offset by compensatory mutations (Poon and Chao 2005). The bulk of putatively deleterious variants occurring in the lowest frequency classes (fig. 1), and a higher level of observed heterozygosity for putatively deleterious variants (supplementary fig. S1, Supplementary Material online) are both consistent with the action of purifying selection on variants with negative impacts on fitness. Putatively disease-causing variants in human populations have also been observed to occur at low frequencies and to occur over a more geographically restricted range (Marth et al. 2011).

Identifying variants with low minor allele frequency is an inexorable part of studying variants with fitness impacts. This presents a problem, as rare variants are the most likely to be affected by false positive variant calls, as they are necessarily observed very few times in the sample. In an attempt to abate the problem of false positive variant calls, we took an iterative approach to variant calling, applying strict genotype quality, read depth, and observed heterozygosity filters to reduce raw variant calls to a high-confidence set of variants. While it is true that some of the variants in our high-confidence set are false positives, they do not dominate our dataset. Our allele frequency spectra (fig. 1), does not show strong skewing of putatively neutral variants toward low-frequency classes, which would be indicative of genotyping errors. In addition, false positive variants are expected to occur randomly, which would lead to roughly equal numbers of first, second, and third position SNPs within codons. Our variant calls show a strong enrichment toward third positions in codons (supplementary fig. S2, Supplementary Material online), which are mostly synonymous positions, and are expected to be neutral. Deficiencies in first and second positions, which are mostly nonsynonymous sites under purifying selection, are indicative of our variant calls consisting mostly of true positive variants.

## Comparison of Identification Methods

Each of the approaches used here to identify deleterious variants makes use of sequence constraint across a phylogenetic relationship. They differ in terms of the models used to assess the functional effect of a variant. SIFT uses a heuristic, which determines if a nonsynonymous variant alters a conserved site based on an alignment built from PSI-BLAST results (Ng 2003). PolyPhen2 is similar, but additionally identifies potential disruptions in secondary or tertiary structure of the encoded protein, when such information is available, (Adzhubei et al. 2010), and is trained on known human disease-causing polymorphisms and neutral polymorphisms. Both of these approaches estimate codon conservation from a multiple sequence alignment, but do not use phylogenetic relationships in their predictions. PolyPhen2 identified the largest number of variants as deleterious, perhaps reflecting bias from the training dataset. Nonhuman systems may differ fundamentally as to which amino acid substitutions tend to have strong functional impact, which would reduce prediction accuracy in other species (Adzhubei et al. 2010). The LRT is a hypothesis-driven approach, and compares the likelihood of two evolutionary scenarios. It explicitly calculates the local synonymous substitution rate, and uses it to test whether an individual codon is under selective constraint or evolving neutrally (Chun and Fay 2009). Variants in selectively constrained codons are considered to be deleterious.

Our results show that even though each prediction approach identifies a similar proportion of nonsynonymous SNPs as deleterious, the overlap between approaches is very small. Because each approach varies slightly in its prediction procedure and assumptions, the intersection of multiple approaches may provide more accurate predictions than any single prediction approach alone. At the genome-wide scale, this pattern is apparent in the frequency distribution of the variants that are identified as deleterious by all three approaches. Variants identified as deleterious by all three approaches are enriched in the lowest frequency class, suggesting that they are under purifying selection (fig. 2). Furthermore known phenotype-altering SNPs are more likely to be predicted to be deleterious by all three approaches than those without known or measurable phenotypic impacts. This suggests that the intersection of prediction approaches tends to identify variants that are more likely to have fitness consequences, especially if the variant has a large effect on a phenotype. Identifying variants that are likely to have large effects on protein function and phenotype improves our ability to identify the nature of trait variation, especially if rare alleles of large effect are major contributors to complex traits (Thornton et al. 2013).

The SNPs predicted to be deleterious differ somewhat between prediction approaches. Even though SIFT and PolyPhen2 identify similar proportions of nonsynonymous SNPs as deleterious, they overlap at only ∼50% of sites (table 2). SNPs identified through at least two approaches seem more likely to be deleterious, based on lower average derived allele frequencies (fig. 1). Comparisons of the distribution of Grantham scores (Grantham 1974) show high similarity in the severity of amino acid replacements that are predicted to be deleterious by each approach (supplementary fig. S4, Supplementary Material online). The effects of reference bias are apparent in SIFT and PolyPhen2. In barley and soybean, the reference genotypes are ‘Morex’ and ‘Williams 82’, respectively. Even when polarized by ancestral and derived alleles, these genotypes show considerably fewer inferred deleterious variants (supplementary tables S6 and S7, Supplementary Material online).

We developed a software package to implement the LRT called BAD_Mutations (BLAST Aligned-Deleterious Mutations). While BAD_Mutations is similar in approach to SIFT and PolyPhen2, it uses distinct data sources and models to predict whether or not a SNP is deleterious. SIFT and PolyPhen2 rely on BLAST searches against a general nucleotide sequence database, which results in high degree of variability in data quality from gene to gene (data not shown). BAD_Mutations, on the other hand, uses a set of assembled and annotated genome sequences available in the public domain in databases such as Phytozome (https://phytozome.jgi.doe.gov, last accessed October 7, 2015) and Ensembl Plants (http://plants.ensembl.org/, last accessed October 7, 2015). The use of a standard set of genome sequences helps to ensure consistent phylogenetic comparisons for each gene analyzed. It also uses a model that weights the conservation of the amino acid residue by the synonymous substitution rate of the gene under consideration (Chun and Fay 2009). BAD_Mutations is open source and freely available at https://github.com/MorrellLAB/BAD_Mutations.

## Rise of Deleterious Variants into Populations

The number of segregating deleterious variants in a species is very different from the number of *de novo* deleterious mutations in each generation, commonly identified as *U*. *U* is the product of the per-base pair mutation rate, the genome size, and the fraction of the genome that is deleterious when mutated (Charlesworth 2012). In humans, *U* is estimated at approximately two new deleterious variants per genome per generation (Agrawal and Whitlock 2012). Estimates from *A.* *thaliana* suggest that the genomic mutation rate for fitness-related traits is 0.1–0.2 per generation (Shaw et al. 2002), approximately half of which are estimated to be deleterious. Even though new mutations are constantly arising, the standing load of deleterious variation greatly exceeds the rate at which they arise (Charlesworth et al. 2004; Charlesworth 2012). However, our results show that ∼40% of our inferred deleterious variants are private to individual cultivars, suggesting that they can be purged from breeding programs.

Once deleterious variants are present as segregating variation in the progenitors of crops, genetic bottlenecks associated with domestication (Eyre-Walker et al. 1998) may allow deleterious variants to drift to higher frequency (Robertson 1960). The selective sweeps associated with domestication and improvement (Wright et al. 2005) would decrease nucleotide diversity in affected genomic regions (Smith and Haigh 1974; Kaplan et al. 1989), and subsequently reduce the effective recombination rate (cf. O’Reilly et al. 2008). The selective and demographic processes of domestication and improvement lead to three basic hypotheses about the distribution of deleterious variants in crop plants: (1) the relative proportion of deleterious variants will be higher in domesticates than in wild relatives; (2) deleterious variants will be enriched near loci of agronomic importance that are subjected to strong selection during domestication and improvement; (3) the relative proportion of deleterious variants will be lower in elite cultivars than landraces due to strong selection for yield (Gaut et al. 2015). Future studies of deleterious variants in crops and their wild relatives can address these hypotheses to understand the source of variation in modern cultivated material.

## Deleterious Variants in Crop Breeding

The identification and targeted elimination of deleterious variants has been proposed as a potential means of improving plant fitness and crop yield (Morrell et al. 2011). Current plant breeding strategies using genome-wide prediction rely on estimating genome-wide marker effects on quantitative traits of interest (Meuwissen et al. 2001). Genome-wide prediction has been shown to be effective in both animals (Schaeffer 2006) and plants (Heffner et al. 2011; Jacobson et al. 2014), but these approaches rely on estimating marker contributions to a quantitative trait (i.e., a measured phenotypic effect). The genetic architecture of these traits suggests that our ability to quantify the effects of individual loci will reach practical limits before we can identify loci contributing to their variance (Rockman 2012). QTL mapping approaches to identifying favorable variants for agronomic traits will reach practical limits, even for variants of large effect (King et al. 2012). Many traits of agronomic interest, particularly yield in grain crops, are quantitative and have a complex genetic basis. As such, they are under the influence of environmental effects and many loci (Falconer and Mackay 1996). Current genome-wide prediction and selection methodologies rely on estimating the combined effects of markers across the genome (Meuwissen et al. 2001), but this approach is limited by recombination rate and the ability to measure phenotypes of interest. The identification and purging of deleterious variants should provide a complementary approach to current breeding methodologies, if bioinformatically identified deleterious variants are truly deleterious (Morrell et al. 2011).

In this study, we restricted our analyses to protein coding regions, though additional recent evidence suggests that deleterious variants can accumulate in conserved noncoding sequences, such as transcription factor binding sites (Arbiza et al. 2013). In addition, insertion and deletion polymorphisms and larger structural variants were not considered in this study. Structural variants are abundant in crop plants, and may be involved with large phenotypic changes (Chia et al. 2012; Anderson et al. 2014). As such, analysis of nonsynonymous SNPs presents a lower bound on the estimates of the number of deleterious variants segregating in populations. Efforts to identify deleterious variants in noncoding sequences are limited by scant knowledge of functional constraints on noncoding genomic regions, and difficulty in aligning noncoding regions from all but the most closely related taxa (Doniger et al. 2008). Annotation of noncoding sequences will uncover additional deleterious variants, but the roughly 1,000 putatively deleterious variants we identify per individual cultivar should provide ample targets for selection of recombinant progeny in a breeding program.

## Materials and Methods

### Plant Material and DNA Sequencing

The exome resequencing data reported here include 13 cultivated barleys, and 2 wild barley accessions. Barley exome capture was based on a 60-Mb liquid-phase Nimblegen capture design (Mascher et al. 2013). For the soybean sample, we resequenced whole genomes of seven soybean cultivars and used previously generated whole genome sequence of *G.* *soja* (Kim et al. 2010). Each sample was prepared and sequenced with manufacturer protocols (Illumina, San Diego, CA) to at least 25× coverage of the target with 76, 100, or 151-bp paired-end reads. A summary of samples and sequencing statistics is given in supplementary table S1, Supplementary Material online.

### Read Mapping and SNP Calling

DNA sequence handling followed the “Genome Analysis Tool Kit (GATK) Best Practices” workflow from the Broad Institute (McKenna et al. 2010; DePristo et al. 2011). Our workflow for read mapping and SNP calling is depicted in supplementary fig. S1, Supplementary Material online. First, reads were checked for proper length, Phred score distribution, and *k*-mer contamination with FastQC (www.bioinformatics.babraham.ac.uk/projects/fastqc/, last accessed June 6, 2014). Primer and adapter sequence contamination was then trimmed from barley reads using Scythe (www.github.com/vsbuffalo/scythe, last accessed April 4, 2014), using a prior on contamination rate of 0.05. Low-quality bases were then removed with Sickle (www.github.com/najoshi/sickle, last accessed October 29, 2014), with a minimum average window Phred quality of 25, and window size of 10% of the read length. Soybean reads were trimmed using the fastqc-mcf tool in the ea-utils package (https://github.com/zachcp/ea-utils, last accessed September 4, 2014). Post-alignment processing and SNP calling were performed with the GATK v. 3.1 (McKenna et al. 2010; DePristo et al. 2011).

Barley reads were aligned to the Morex draft genome sequence (Mayer et al. 2012) using BWA-MEM (Li and Durbin 2009). We tuned the alignment reporting parameter and the gapping parameters to allow ∼2% mismatch between the reads and reference sequence, which is roughly equivalent to the highest estimated nucleotide diversity observed at a locus in barley CDS (Morrell et al. 2003, 2006, 2014). The resulting SAM file was trimmed of unmapped reads with SAMtools version 0.1.18 (Li et al. 2009), sorted, and trimmed of duplicate reads with Picard version 1.118 (http://broadinstitute.github.io/picard/, last accessed July 28, 2014). We then realigned around indels, using a set of 100 previously known indels from Sanger resequencing of 25 loci (Caldwell et al. 2006; Morrell and Clegg 2007; Morrell et al. 2014). Sequence coverage was estimated with ‘bedtools genomecov,’ using the regions included in the Nimblegen barley exome capture design (https://sftp.rch.cm/diagnostics/sequencing/nimblegen_annotations/ez_barley_exome/barley_exome.zip, last accessed December 20, 2013) and bedtools version 2.20.0. Individual sample alignments were then merged into a multisample alignment for variant calling. A preliminary set of variants was called with the GATK HaplotypeCaller with a heterozygosity (average pairwise diversity) value of 0.008, based on average CDS diversity reported for cultivated barley (Morrell et al. 2014). This preliminary set of variants was filtered to sites with a genotype score of 40 or greater, heterozygous calls in at most two individuals, and read depth of at least five reads. We then used the filtered variants, SNPs identified in the Sanger resequencing dataset, and 9,605 SNPs from genotyping assays: 5,010 from the James Hutton Institute (Comadran et al. 2012), and 4,595 from Illumina GoldenGate assays (Close et al. 2009) as input for the GATK VariantRecalibrator to obtain a set of recalibrated variant calls. Final variants were filtered to be supported by a minimum of five reads per sample, have a Phred-scaled genotype quality of at least 40, and have a maximum of two accessions with missing data.

Processing of soybean samples is as described above, but with the following modifications. Soybean reads were aligned to the Williams 82 reference genome sequence (Schmutz et al. 2010). Mismatch and reporting parameters for the cultivated samples were adjusted to allow for ∼1% mismatch between reads and reference, which is approximately the highest CDS diversity typically observed in soybean (Hyten et al. 2006). The alignments were trimmed and sorted as described above. Preliminary variants were called as in the barley sample, but with a heterozygosity value of 0.001, which is the average nucleotide diversity reported by Hyten et al. (2006). Final variant calls were obtained in the same way as described for the barley sample, using SNPs on the SoySNP50K chip (Song et al. 2013) as known variants.

Transition to transversion ratios were calculated with R scripts. The ratios in the Sanger resequencing dataset were computed using SNPs identified in FASTA alignments of wild barley gene sequences (Morrell et al. 2006), or a table of SNPs identified in resequencing of soybean gene fragments (supplemental data file 1 in Hyten et al. 2006).

Read mapping scripts, variant calling scripts, and variant filtering scripts for both barley and soybean are available on GitHub at (www.github.com/MorrellLAB/Deleterious_Mutations).

### SNP Classification

Barley SNPs were identified as coding or noncoding using the Generic Feature Format v3 (GFF) file provided with the reference genome (Mayer et al. 2012). A custom Python script was then used to identify coding barley SNPs as synonymous or nonsynonymous. Soybean SNPs were assigned using primary transcripts using the Variant Effect Predictor (VEP) from Ensembl (www.ensembl.org/info/docs/tools/vep/index.html). Nonsynonymous SNPs were then assessed using SIFT (Ng 2003), PolyPhen2 (Adzhubei et al. 2010) using the ‘HumDiv’ model, and an LRT comparing codon evolution under selective constraint to neutral evolution (Chun and Fay 2009). For the likelihood ratio test, we used the phylogenetic relationships between 37 Angiosperm species based on genic sequence from complete plant genome sequences available through Phytozome (https://phytozome.jgi.doe.gov, last accessed October 7, 2014) and Ensembl Plants (http://plants.ensembl.org/, last accessed October 7, 2014). The LRT is implemented as a Python package we call ‘BAD_Mutations’ (www.github.com/MorrellLAB/BAD_Mutations). CDSs from each genome were downloaded and converted into BLAST databases. The CDS from the query species was used to identify the best match from each species using TBLASTX. The best match from each species was then aligned using PASTA (Mirab et al. 2014), a phylogeny-aware alignment tool. The resulting alignment was then used as input to the LRT for the affected codon. The LRT was performed on codons with a minimum of 10 species represented in the alignment at the queried codon. Reference sequences were masked from the alignment to reduce the effect of reference bias (Simons et al. 2014). A SNP was identified as deleterious if the *P*-value for the test was <0.05, with a Bonferroni correction applied based on the number of tested codons, and if either the alternate or reference allele was not seen in any of the other species. For barley, our threshold was 8.4E−7 (59,277 codons tested), and for soybean, our threshold was 7.8E−7 (64,087 codons tested). A full list of species names and genome assembly and annotation versions used is available in supplementary table S9, Supplementary Material online.

### Relating Recombination Rate to Deleterious Predictions

Recombination rates were taken from a genetic map developed by Lee et al. (2015). In brief, a recombinant inbred line family was derived from a cross between a wild soybean line and a cultivated soybean line, and genotyped with the SoySNP6K genotyping platform. For our analysis, we calculated cM/Mb values for each interval between markers on the SoySNP6K. Within each interval, we also calculated the proportion of nonsynonymous SNPs that annotated as deleterious by our criteria. Intervals with negative, or cM/Mb values >20, were excluded, as they indicate regions where the markers likely have incorrect physical position. Pearson correlation (supplementary fig. S3*A*, Supplementary Material online) and logistic regression (supplementary fig. S3*B*, Supplementary Material online) were used to investigate the relationship between recombination rate and deleterious variation.

### Inference of Ancestral State

Prediction of deleterious mutations is complicated by reference bias (Chun and Fay 2009; Simons et al. 2014), which manifests in two ways. First, individuals that are closely related to the strain used for the reference genome will appear to have fewer genetic variants, and thus fewer inferred nonsynonymous and deleterious variants. Second, when the reference strain carries a derived allele at a polymorphic site, that site is generally not predicted to be deleterious (Simons et al. 2014). To address the issue of reference bias, we polarized all coding variants by ancestral and derived state, rather than reference and nonreference state. Ancestral states were inferred for SNPs in gene regions by inferring the majority state in the most closely related clade from the consensus phylogenetic tree for the species included in the LRT. For barley, the ancestral states were inferred from gene alignments of *Aegilops tauschii*, *Brachypodium distachyon*, and *Tritium urartu*. For soybean, ancestral states were inferred using *Medicago truncatula* and *Phaseolus vulgaris*. This approach precludes universal inference of ancestral state for noncoding variants. However, examination of alignments of intergenic sequence in *Triticeae* species and in *Glycine* species showed that alignments outside of CDS is not reliable for ancestral state inference (data not shown).

## Supplementary Material

Supplementary figures S1–S6 and tables S1–S9 are available at *Molecular Biology and Evolution* online (http://www.mbe.oxfordjournals.org/).

Supplementary Data
