# Supplementary material for: The Role of Deleterious Substitutions in Crop Genomes
Source: Mol Biol Evol. 2016 Jun 14;33(9):2307–17. doi: 10.1093/molbev/msw102 (PMC4989107; doi:10.1093/molbev/msw102)
Supplement: Supplementary Data [file supp_33_9_2307__index.html]

The Role of Deleterious Substitutions in Crop Genomes — Supplementary Data 

# The Role of Deleterious Substitutions in Crop Genomes

## Supplementary Data

files

- Supplementary Data - zip file
