# Supplementary material for: The Role of Deleterious Substitutions in Crop Genomes
Source: Mol Biol Evol. 2016 Jun 14;33(9):2307–17. doi: 10.1093/molbev/msw102 (PMC4989107; doi:10.1093/molbev/msw102)
Supplement: Supplementary Data [file supp_msw102_suppl_data.zip › Figure S4.pdf]

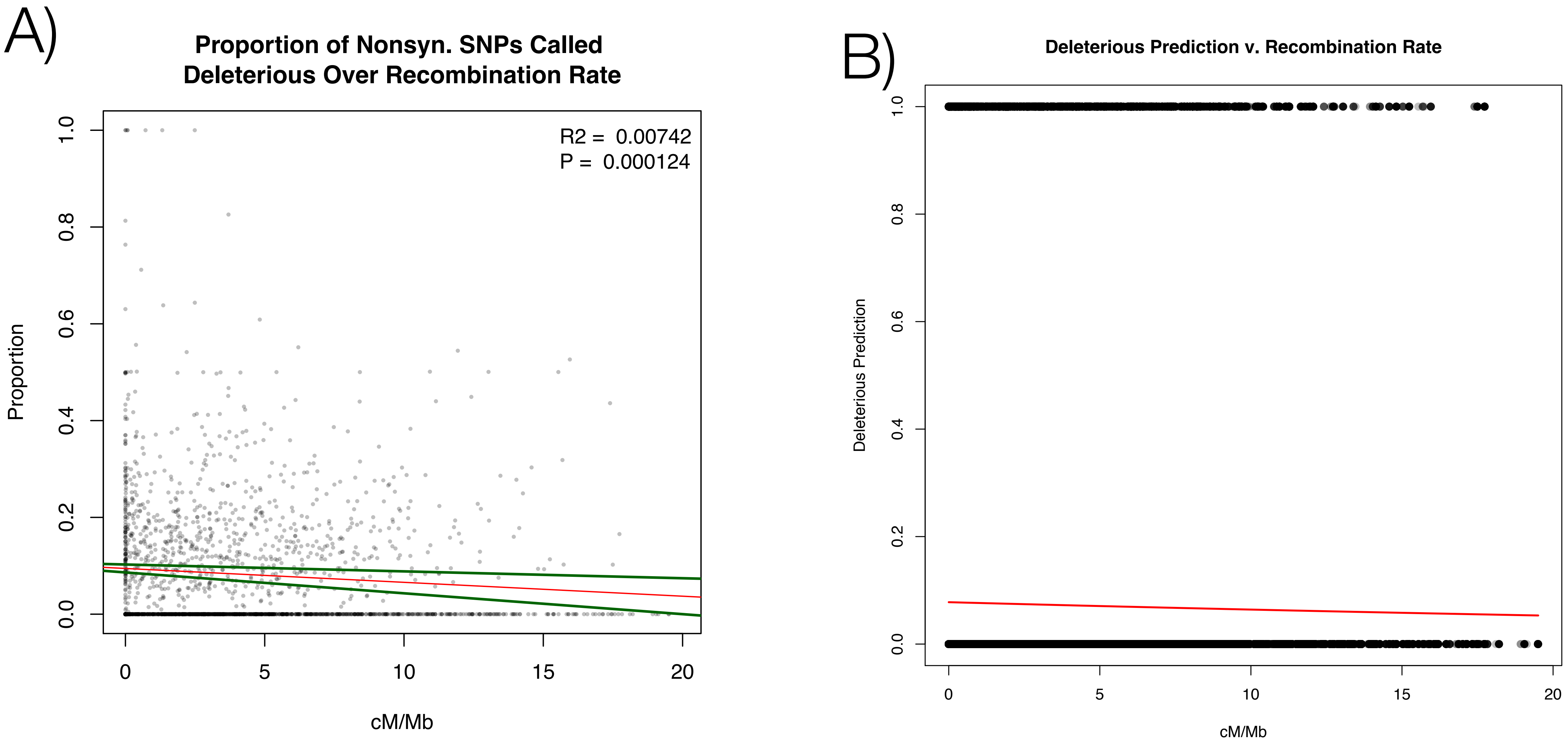

A)
Proportion of Nonsyn. SNPs Called Deleterious Over Recombination Rate
R2 = 0.00742
P = 0.000124
Proportion
cM/Mb
0.0
0.2
0.4
0.6
0.8
1.0
0
5
10
15
20
B)
Deleterious Prediction v. Recombination Rate
Deleterious Prediction
cM/Mb
0.0
0.2
0.4
0.6
0.8
1.0
0
5
10
15
20
