# Supplementary figures and images for: The Role of Deleterious Substitutions in Crop Genomes
Source: Mol Biol Evol. 2016 Jun 14;33(9):2307–17. doi: 10.1093/molbev/msw102 (PMC4989107; doi:10.1093/molbev/msw102)

# Distribution of Heterozygosity for Deleterious and Tolerated SNPs

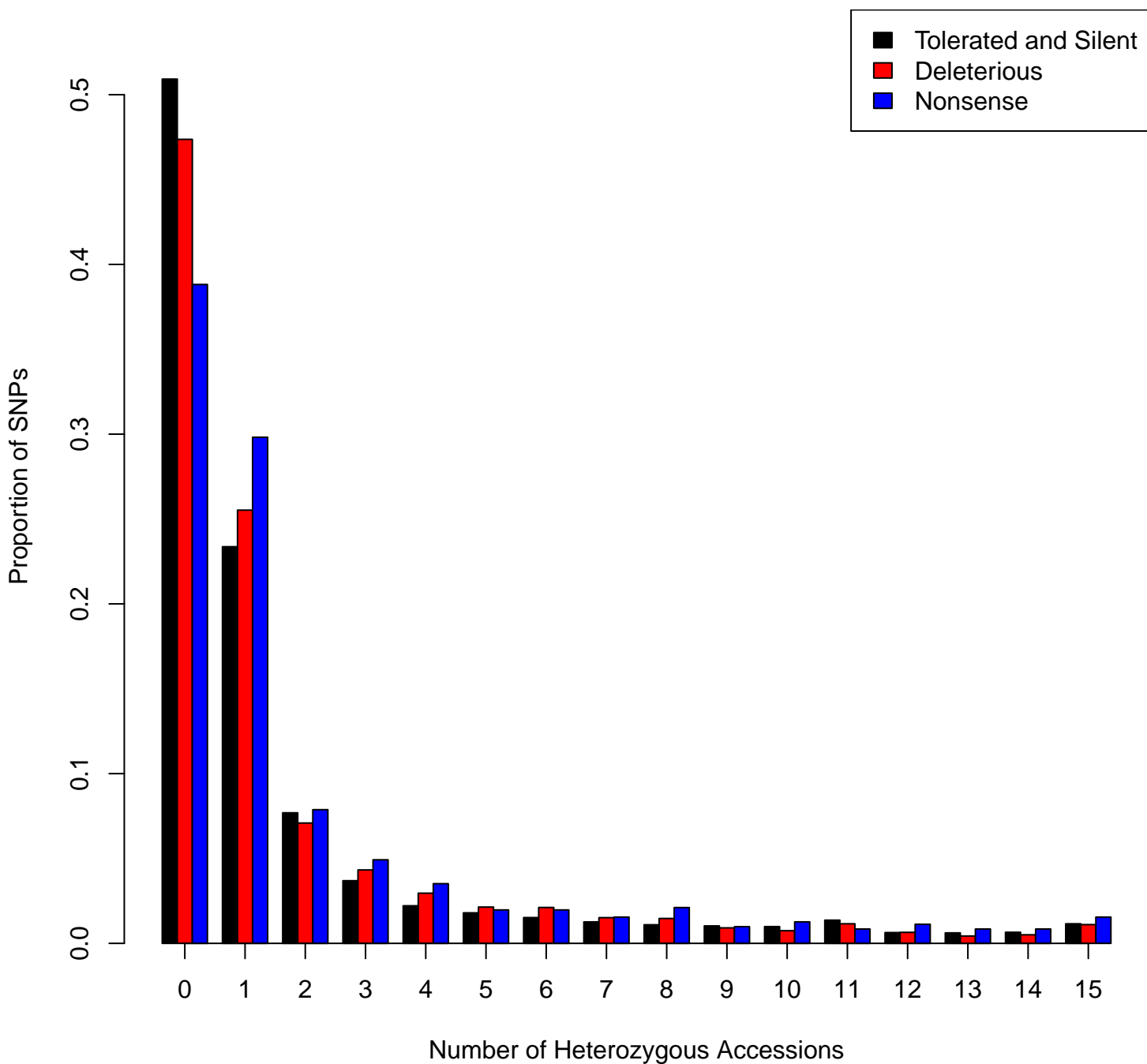

Supplement: Supplementary Data [file supp_msw102_suppl_data.zip › Figure S1.pdf]

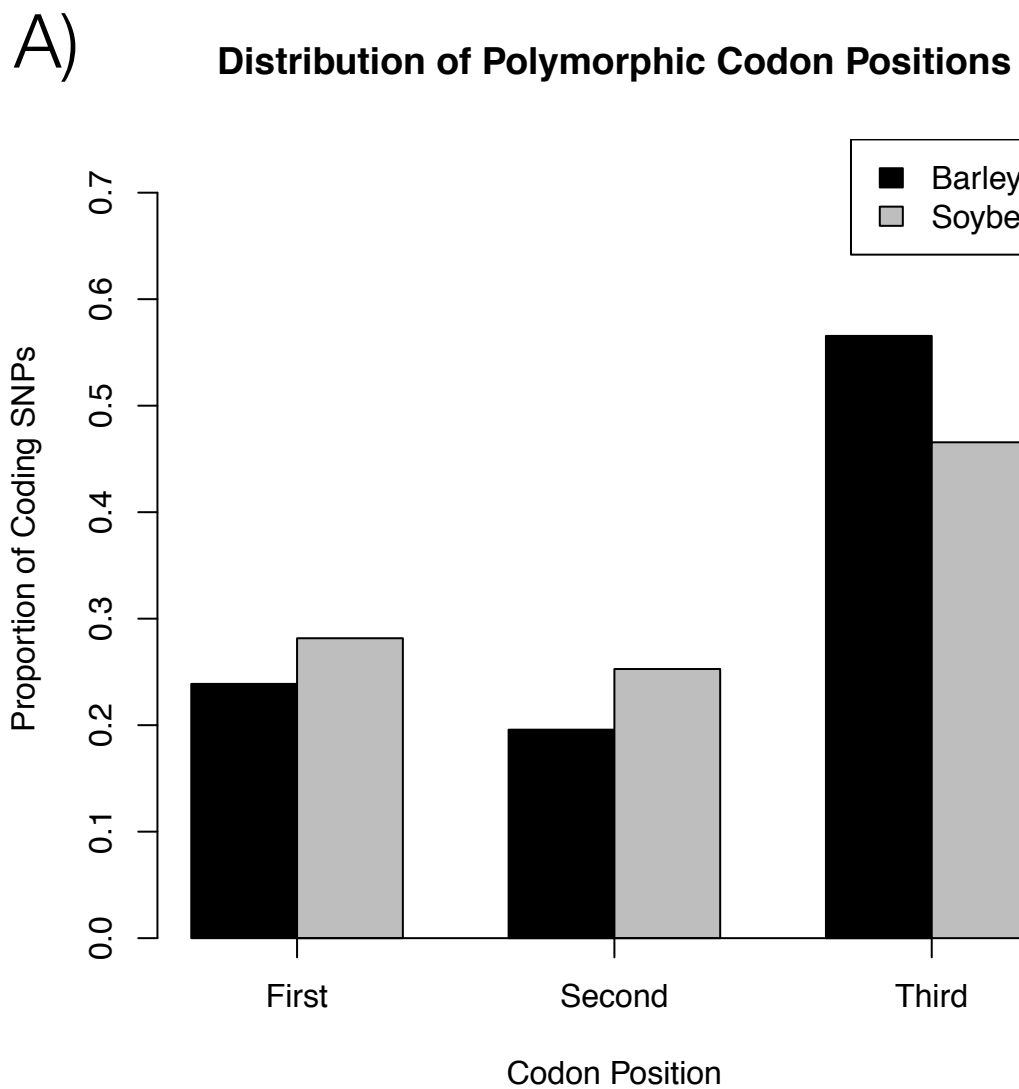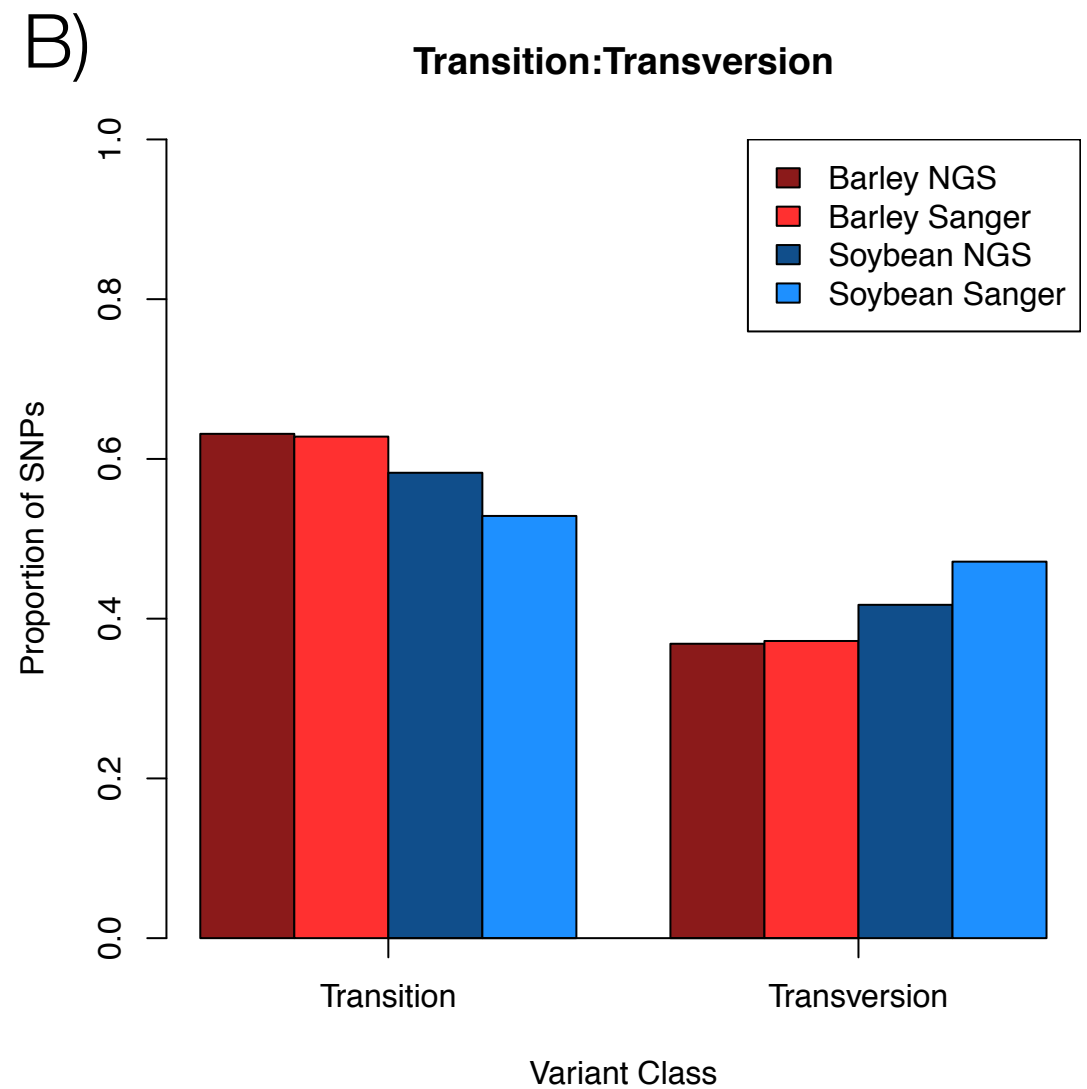

Supplement: Supplementary Data [file supp_msw102_suppl_data.zip › Figure S2.pdf]

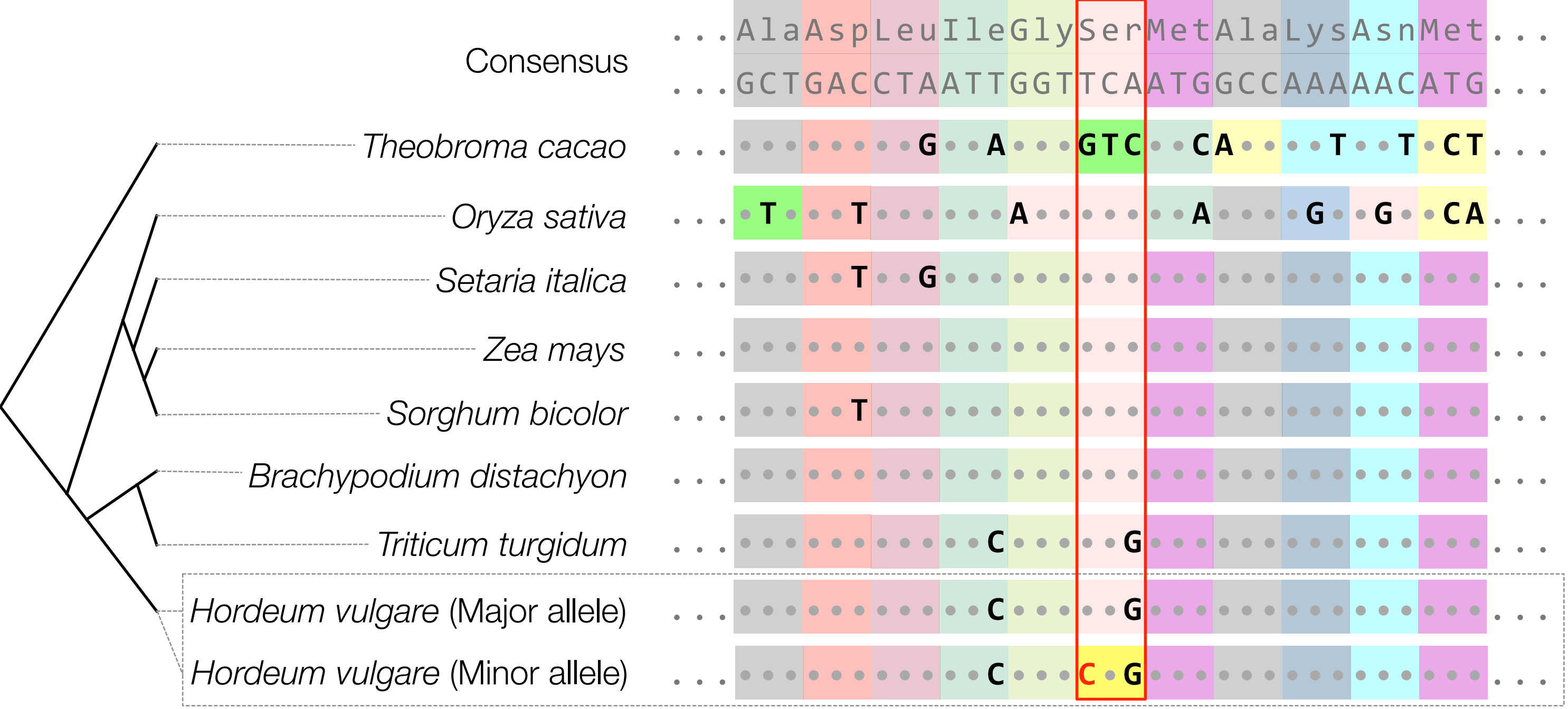

Supplement: Supplementary Data [file supp_msw102_suppl_data.zip › Figure S3.pdf]

**Distribution of Grantham Scores**

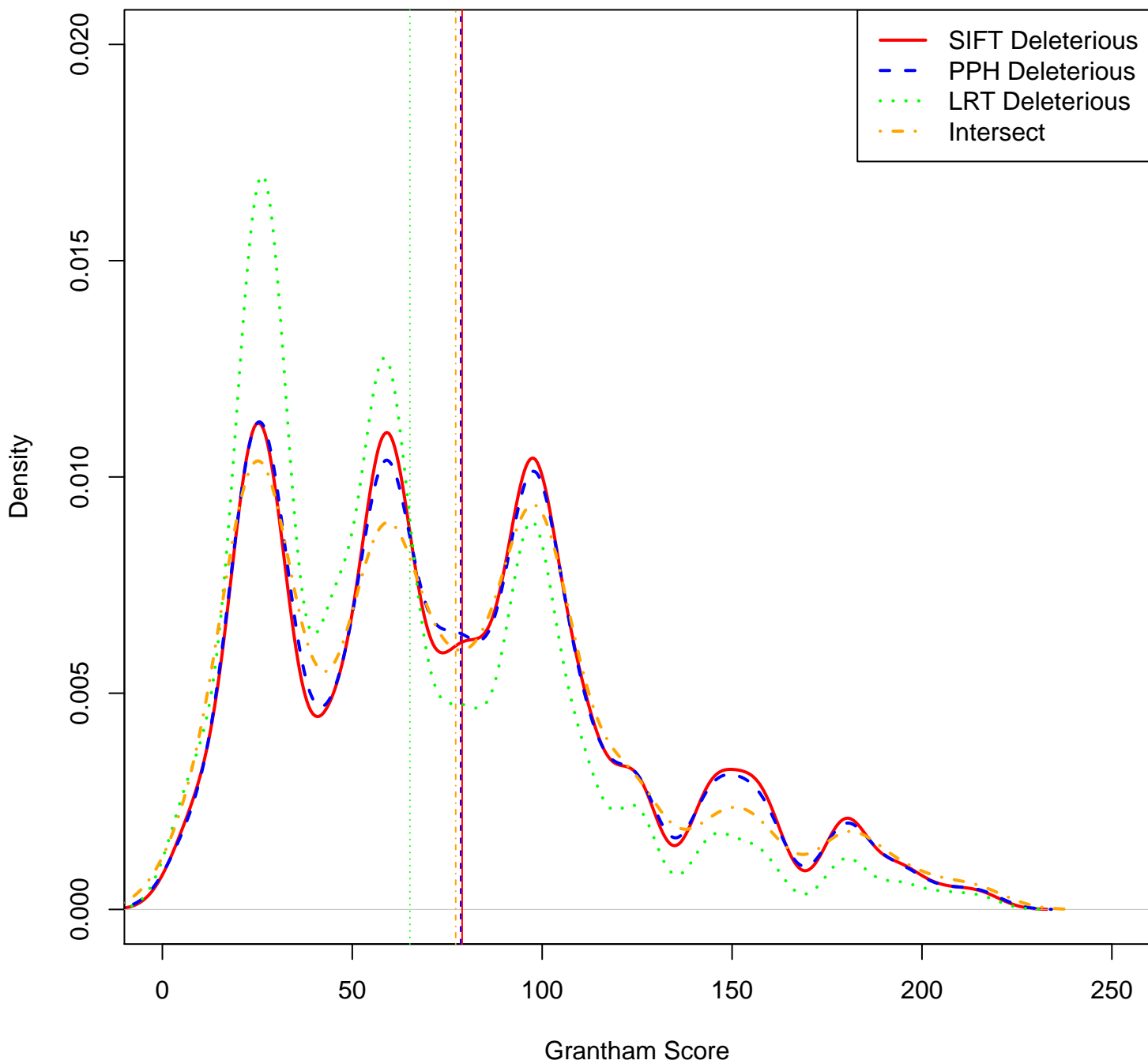

Supplement: Supplementary Data [file supp_msw102_suppl_data.zip › Figure S5.pdf]

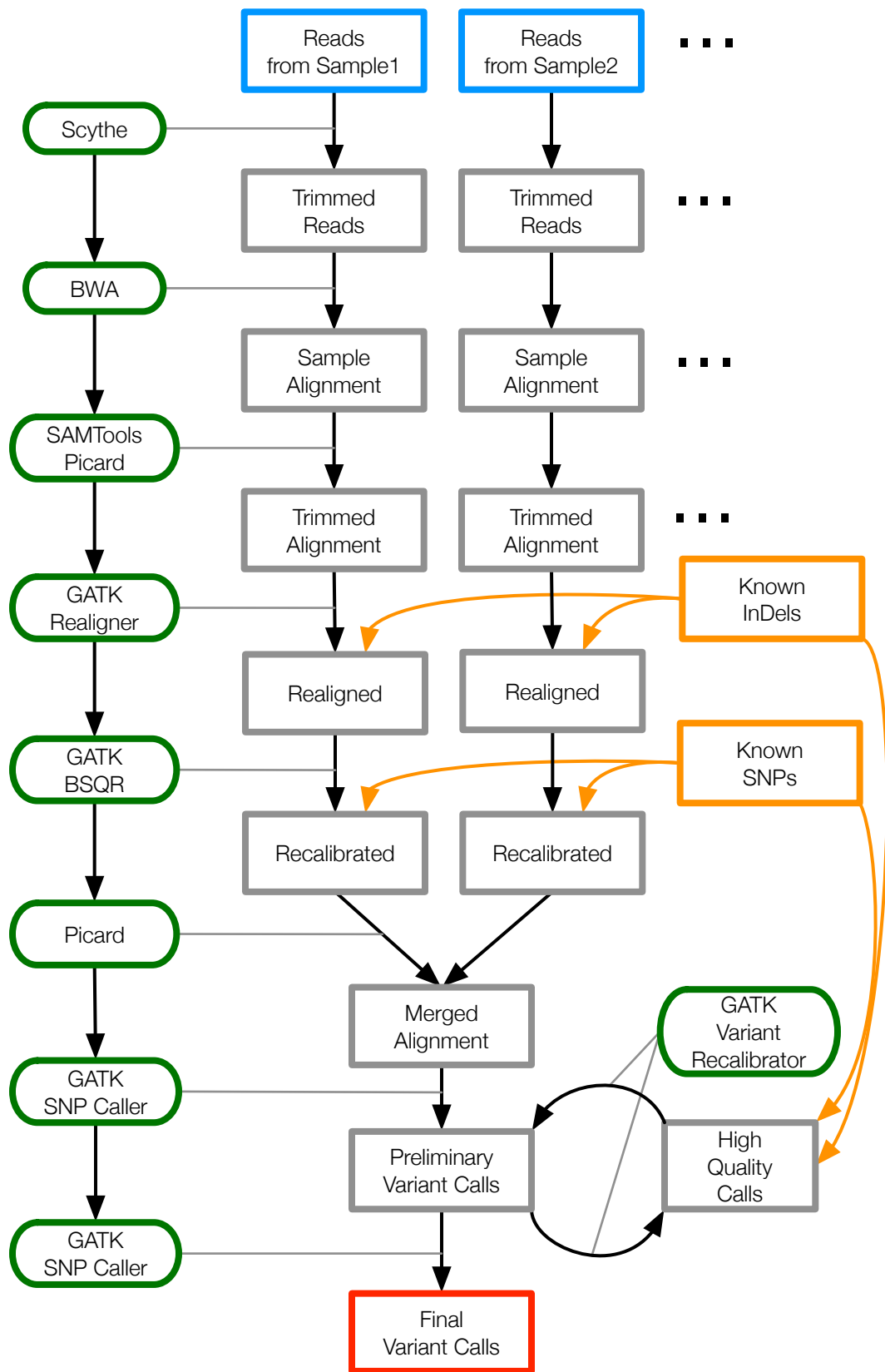

Supplement: Supplementary Data [file supp_msw102_suppl_data.zip › Figure S6.pdf]
